# Pyramiding of drought yield QTLs into a high quality Malaysian rice cultivar MRQ74 improves yield under reproductive stage drought

**DOI:** 10.1186/s12284-016-0093-6

**Published:** 2016-05-10

**Authors:** Noraziyah Abd Aziz Shamsudin, B. P. Mallikarjuna Swamy, Wickneswari Ratnam, Ma. Teressa Sta. Cruz, Nitika Sandhu, Anitha K. Raman, Arvind Kumar

**Affiliations:** Faculty of Science and Technology, Universiti Kebangsaan Malaysia, 43600 Bangi, Selangor Malaysia; Plant Breeding, Genetics, and Biotechnology Division, International Rice Research Institute, DAPO Box 7777 Metro Manila, Philippines

**Keywords:** Drought, Drought yield QTLs, Marker assisted breeding, Pyramiding, Rice

## Abstract

**Background:**

With the objective of improving the grain yield (GY) of the Malaysian high quality rice cultivar MRQ74 under reproductive stage drought stress (RS), three drought yield QTLs, viz. *qDTY*_2.2_, *qDTY*_*3.1*_, and *qDTY*_*12.1*_ were pyramided by marker assisted breeding (MAB). Foreground selection using QTL specific markers, recombinant selection using flanking markers, and background selections were performed in every generation. BC_1_F_3_ derived pyramided lines (PLs) with different combinations of *qDTY*_2.2_, *qDTY*_*3.1*_, and *qDTY*_*12.1*_ were evaluated under both RS and non-stress (NS) during the dry season (DS) of 2013 and 2014 at IRRI.

**Results:**

The GY reductions in RS trials compared to NS trials ranged from 79 to 99 %. Plant height (PH) was reduced and days to flowering (DTF) was delayed under RS. Eleven BC_1_F_5_ MRQ74 PLs with yield advantages of 1009 to 3473 kg ha^−1^ under RS and with yields equivalent to MRQ74 under NS trials were identified as promising drought tolerance PLs. Five best PLs, IR 98010-126-708-1-4, IR 98010-126-708-1-3, IR 98010-126-708-1-5, IR 99616-44-94-1-1, and IR 99616-44-94-1-2 with a yield advantage of more than 1000 kg ha^−1^ under RS and with yield potential equivalent to that of MRQ74 under NS were selected. The effect of three drought grain yield QTLs under RS in MRQ74 was validated. Under NS, PLs with two *qDTY* combinations (*qDTY*_*2.2* +_*qDTY*_*12.1*_) performed better than PLs with other *qDTY* combinations, indicating the presence of a positive interaction between *qDTY*_*2.2*_ and *qDTY*_*12.1*_ in the MRQ74 background.

**Conclusion:**

Drought tolerant MRQ74 PLs with a yield advantage of more than 1000 kg ha^−1^ under RS were developed. Differential yield advantages of different combinations of the *qDTYs* indicate a differential synergistic relationship among *qDTYs*.

## Background

World rice production has tripled from 221 million tons in 1961 to more than 670 million tons in 2010 (FAOSTAT 2013). The expansion of rice growing areas and the adoption of high-yielding semi-dwarf varieties are important factors that contribute to increased rice production. However, most semi-dwarf high-yielding varieties show increased susceptibility to drought (Vikram et al. 2011), a phenomenon which is predicted to escalate in both rainfed as well as irrigated ecosystems in the future (IRRI 2012).

Nearly 27 million ha of rainfed rice are frequently affected by drought (IRRI 2012). The yield loss to drought is estimated at 13 to 35 % in Thailand and more than USD 3 million worth of losses were incurred in Malaysia in 2009 (Anon 2010). The susceptibility of the rice crop to water stress is more critical at its reproductive stages, where moderate to severe stress during flowering may result in huge reductions in rice yield (Garrity and O’ Toole 1994; Lafitte et al. 2004; Atlin et al. 2006; Barnabás et al. 2007), as experienced by nearly 70 % of lowland rice farmers in sub-Saharan Africa (Sié et al. 2008). Drought poses a threat in the need to increase the global production of rice by 70 % in the next 35 years in order to meet the growing food demand (Cooper and Hammer 1996). Most of this increase has to come from rainfed lowland and upland rice ecosystems. Therefore, breeding for drought tolerance varieties needs to become a priority for rice breeders in Asia and Africa.

Several traditionally grown landraces such as Khao Dawk Mali, Azucena, Dular, Rayada, Bala, Apo, Nam Sagui 19, Nagina 22, Aday Sel, Dehula, Moroberekan, Huma Wangi Lenggong, Siam Pilihan, Chianung Sen Yu, Kashmir Basmati, and MR142 as well as some wild species rice accessions have great adaptability to drought, and their hidden genetic potential offers a better opportunity to improve drought tolerance in mega-varieties (Pantuwan et al. 2002; Venuprasad et al. 2009; Venuprasad et al. 2007; Henry et al. 2011; Vikram et al. 2012; Swamy and Kumar 2013). These traditional donors possess genes/QTLs for better ability to tolerate drought than high-yielding semi-dwarf varieties. However, most of these donors have low yield potential and other undesirable traits. Several studies have been conducted to generate improved drought tolerance pre-breeding lines by crossing traditional drought-tolerant donors with drought-susceptible mega-varieties (Swamy and Kumar 2013).

Research at the International Rice Research Institute (IRRI) during the last few years has led to the identification of several major drought grain yield QTLs (*qDTYs*) under drought (Bernier et al. 2007; Venuprasad et al. 2009; Vikram et al. 2011; Ghimire et al. 2012; Mishra et al. 2013; Yadaw et al. 2013; Dixit et al. 2014). These studies found that lines with *qDTYs* produced higher grain yield (GY) than most of the traditional and modern rice accessions from Malaysia under reproductive stage drought stress (RS) condition (Noorzuraini 2013).

The development of drought tolerance varieties could be made more efficient through the introgression of drought yield QTLs through marker assisted breeding (MAB). This approach has been successfully proven with Vandana lines introgressed with *qDTY*_*12.1*_ which produced about 500 kg ha^−1^ yield advantage over its donor parent under RS, and had similar yield to Vandana under non-stress (NS) condition (Kumar et al. 2014). Consistent efforts have been made to introgress and pyramid the identified *qDTYs* into the drought-susceptible mega-variety IR64 through MAB (Swamy et al. 2013). Generally, the identified major effect *qDTYs* have a yield gain of 10 to 30 % and a yield advantage of 150 to 500 kg ha^−1^. However, to provide significant economic benefit to farmers, a yield advantage of at least 1000 kg ha^−1^ is required. For this, pyramiding two or more *qDTYs* showing positive interactions has been suggested as an efficient approach (Swamy and Kumar 2013). However, the effect of identified *qDTYs* in diverse genetic backgrounds remains unknown. Furthermore, very limited studies have been reported which worked on improving high quality rice cultivars under RS. In this study, three *qDTYs,* namely *qDTY*_*2.2*_, *qDTY*_*3.1*_, and *qDTY*_*12.1*_ were pyramided through marker assisted QTL pyramiding into the high quality rice cultivar MRQ74 from Malaysia to improve its yield under drought. The study also aimed to understand the interactions between different *qDTY* combinations and to identify promising drought-tolerant MRQ74 pyramiding lines (PLs).

## Results

### Selection of the donor and recipient parents

Three *qDTYs* were pyramided by marker assisted QTL pyramiding into the high quality rice cultivar MRQ74 from Malaysia. Three drought-tolerant improved lines developed at IRRI, namely IR 84984-83-15-18-B, IR 77298-14-1-2-10, and IR 81896-B-B-195 were used as donors for QTLs *qDTY*_*12.1*_, *qDTY*_*2.2*_, and *qDTY*_*3.1*_, respectively.

### Development of BC_1_F_3_ pyramided lines using marker-assisted breeding

**Fig. 1:**
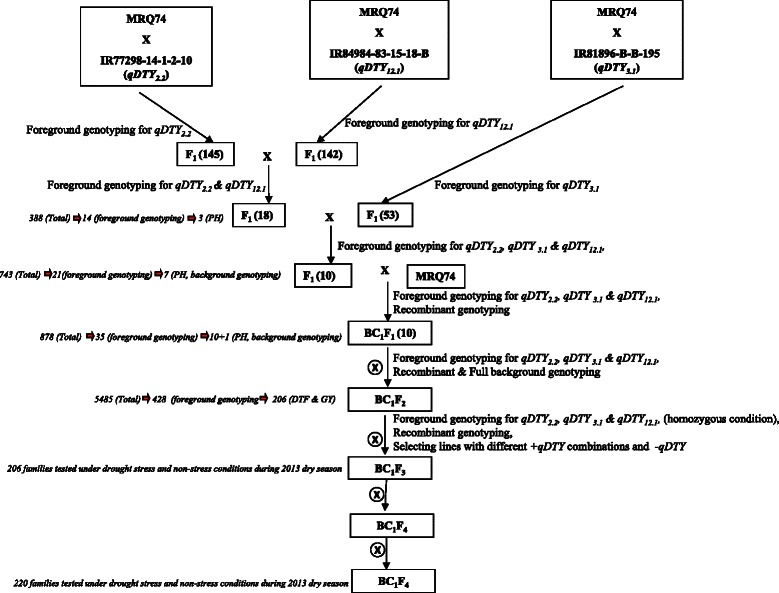
Crossing scheme for the development of BC_1_F_5_ MRQ74 pyramided lines and number of plants selected in every generation

Intercross followed by backcross strategy was used to develop the MRQ74 PLs. The scheme of developing the MRQ74 PLs and number of selected plants in every generation is shown in Fig. 1. The F_1_s from the crosses of MRQ74/IR 77298-14-1-2-10, MRQ74/IR 81896-B-B-195, and MRQ74/IR 84984-83-15-18-B were confirmed for the presence of the QTLs using *qDTY*-specific peak simple sequence repeat (SSR) markers (RM12460 for *qDTY*_*2.2*_, RM520 for *qDTY*_*3.1*_, and RM511 for *qDTY*_*12.1*_). Around 94 to 96 % of the total F_1_ seeds from all crosses amplified alleles of both parents (heterozygous), indicating their true hybrid natures. Five true and confirmed vigorous F_1_ plants from MRQ74/IR 77298-14-1-2-10 and 20 true and vigorous F_1_ plants from MRQ74/IR 84984-83-15-18-B were intercrossed to develop the F_1(2)_ generation with two *qDTYs* (*qDTY*_*2.2*_ and *qDTY*_*12.1*_). A new set of 388 F_1(2)_ seeds was produced from the crosses between F_1_ plants from MRQ74/IR 77298-14-1-2-10 and F_1_ plants from MRQ74/IR 84984-83-15-18-B, and the new set of F_1(2)_ plants was genotyped with peak and other foreground SSR markers of *qDTY*_*2.2*_ and *qDTY*_*12.1*_ loci (RM154, RM233A, RM12460, RM279, RM12569, RM28048, RM28099, RM28130, RM511, RM1261, RM28172 and CG29430). However, only 14 plants (3.6 %) showed donor alleles in two *qDTY* regions. 53 confirmed F_1_ plants from the cross of MRQ74/IR 84984-83-15-18-B were crossed with 14 selected F_1(2)_ plants with two *qDTYs* (*qDTY*_*2.2*_ + *qDTY*_*12.1*_) to produce a large number of F_1(3)_ seeds with all three *qDTYs* (*qDTY*_*2.2*_, *qDTY*_*3.1*_, and *qDTY*_*12.1*_). Three F_1(3)_ individual plants (IR 97978, IR 97982, and IR 97989) that had morphological characteristics similar to MRQ74 were grown during the 2011 wet season (WS).

During the 2011 WS, 734 F_1(3)_ plants from seeds of the three plants (IR 97978, IR 97982, and IR 97989) were genotyped using three peak markers. Based on this initial genotyping, plants possessing the peak markers were selected and all the remaining foreground SSR markers were ran on selected plants. Finally, 21 plants with all the three *qDTY* specific alleles in all the marker loci and the recipient parent at the QTL flanking loci were identified. Out of the 21 plants, 7 plants were morphologically similar to MRQ74 and were backcrossed to MRQ74 to obtain the BC_1_F_1_ seeds.

In the 2012 dry season (DS), 878 BC_1_F_1_ plants from eight BC_1_F_1_s (IR99616, IR98006, IR98007, IR99787, IR98008, IR98010, IR98011, and IR98012) were genotyped with three peak markers. The same foreground selection procedure followed in the F_1(3)_ generation was also followed in the BC_1_F_1_ generation. Finally, only 10 BC_1_F_1_ plants which were similar to MRQ74 were selected after foreground genotyping. The selected BC_1_F_1_ plants were genotyped with 48 polymorphic background markers, with the background recovery varying from 82 to 90 %. The selected BC_1_F_1_ plants were selfed to produce the BC_1_F_2_ generation.

In the 2012 WS, a total of 5485 BC_1_F_2_ plants derived from 10 different BC_1_F_1_ plants were grown and genotyped. There were 428 BC_1_F_2_ plants that were homozygous at different *qDTYs* and their combinations, but only 206 BC_1_F_2_ plants with different *qDTY* and their combinations were selected based on their morphological similarity to MRQ74. These 206 BC_1_F_2_ plants were selfed to obtain their BC_1_F_3_ seeds and further advanced to develop *qDTY* MRQ74 PLs.

### Imposition of drought stress

**Fig. 2:**
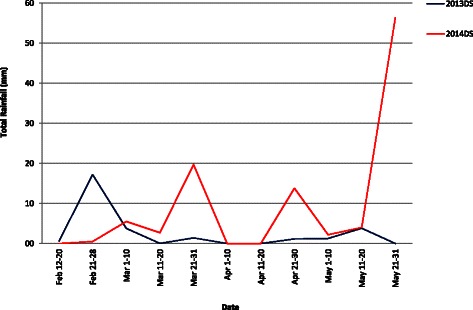
Total cumulative rainfall during reproductive stage of the crop during 2013 and 2014 dry season

**Fig. 3:**
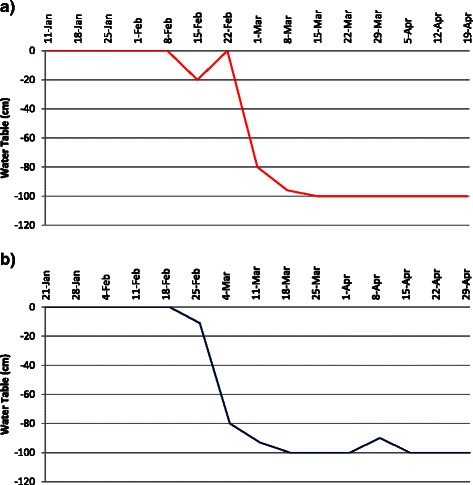
Parching water table in stress trials; (**a**) during 2013 dry season; (**b**) during 2014 dry season

**Fig. 4:**
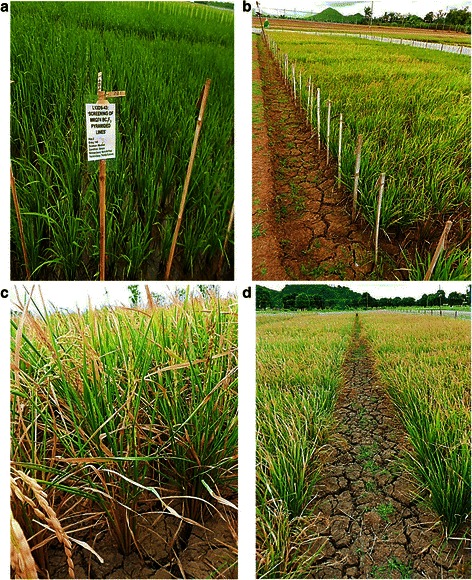
Crop at various stages in the RS trial after stress imposition: (**a**) after 3 weeks of transplanting; (**b**) at reproductive stage; (**c**) at severe stress showing leaf rolling; (**d**) at maturity stage

Daily rainfall data at the IRRI experimental field during the months of February to May in the 2013 DS and 2014 DS was recorded (Fig. 2). In the 2013 DS planting season, the total rainfall was 29.2 mm and the RS treatments was initiated in the 2^nd^ week of February. The stress trials were not irrigated starting from February 8 but received 3.8 mm rainfall in the 1^st^ week of March. From the 2^nd^ week of March up to the 1^st^ week of May, the stress trials received only 1.4 mm rainfall. Rainfall on the 3^rd^ week of February disturbed the stress but againthe ground water table reached 100 cm below the ground after three weeks and continued so until harvest, indicating that the crop faced severe RS during flowering stage (Fig. 3). In the 2014 DS, the stress trials were not irrigated starting from February 19 until harvesting. However, the stress trials received 0.5 to 19.7 mm rainfall in the first four weeks when the stress was imposed. Ground water table continued to decrease up to 73 cm within a week in 2014 DS. In both seasons, the average water table depth during critical flowering stage was 100 cm. Figure 4 shows the crop at various stages in the RS trials after stress imposition.

### Validation of marker assisted breeding for drought tolerance by phenotyping

#### MRQ PL means and trial heritability

The average days to flowering (DTF) in the MRQ74 PLs varied from 86 to 93 days in the NS trials and 91 to 99 days in the RS trials (Table 1). Compared to the NS trials, drought delayed flowering by 4 to 7 days in the RS trials. Plant height (PHT) ranged from 54 to 68 cm in the RS trials and from 82 to 85 cm in the NS trials (Table 1). RS reduced plant height by 17 to 28 cm. Grain yield (GY) ranged from 374 to 836 kg ha^−1^ in the RS experiments and from 3768 to 5680 kg ha^−1^ in the NS experiments (Table 1). The 79 to 99 % reduction in yield under RS as compared to NS showed that the MRQ74 PLs were subjected to severe RS in both seasons. The heritability (*H*) of DTF for the MRQ74 PLs in the RS and NS trials was high (0.71 to 0.89) and was medium to high for both plant height (0.57 to 0.87) and GY (0.48 to 0.84) in both RS and NS trials.

**Table 1:** Means for days to flowering (DTF), plant height (PHT), and grain yield (GY) of MRQ74 PLs as compared to MRQ74 under lowland reproductive stage drought stress and non-stress conditions

Season/Year	Stress	Duration	No. of MRQ 74 PLs	DTF			PHT (cm)			GY (kg ha^−1^)		
				Mean		H	Mean		H	Mean		H
				MRQ74 PLs	MRQ 74		MRQ74 PLs	MRQ 74		MRQ74 PLs	MRQ 74	
DS2013	Non-stress	Medium	130	86 ± 3.8	94	0.88	83 ± 4.28	85	0.87	3768 ± 835.4	4302	0.75
DS2013	Drought	Medium	130	91 ± 3.79	98	0.89	58 ± 4.9	55	0.66	774 ± 267.5	55	0.84
DS2013	Non-stress	Late	102	93 ± 4.7	89	0.71	85 ± 3.9	82	0.74	4585 ± 1003.2	4044	0.56
DS2013	Drought	Late	102	97 ± 4.2	93	0.72	68 ± 4.2	65	0.57	836 ± 820.8	579	0.48
DS2014	Non-stress	Late	235	92 ± 3.7	96	0.88	82 ± 4.0	78	0.84	5680 ± 932.3	5104	0.81
DS2014	Drought	Late	235	99 ± 5.5	105	0.79	54 ± 4.5	55	0.63	374 ± 373	169	0.77

*DS* dry season, *DTF* days to 50 % flowering, *PHT, in cm* and plant height, *M* means ± SE, *H* broad-sense heritability, *GY* grain yield, in kg ha^−1^

### Performance of different combinations of qDTY

**Fig. 5:**
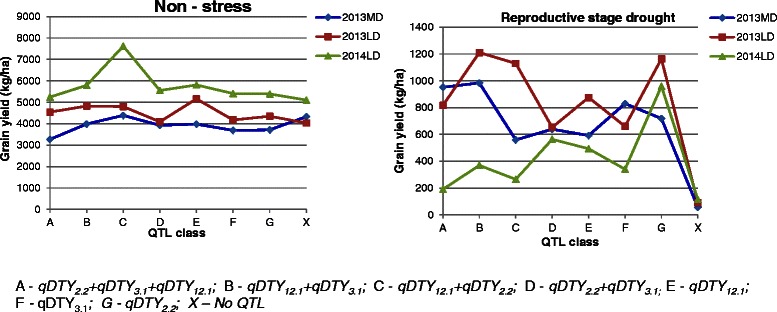
Graph representing QTL classes (X axis) and mean grain yield (Y axis) of medium (MD) and long duration (LD) lines with MRQ74 as the recipient parent. Trials were conducted during dry seasons of years 2013 and 2014. MD- medium duration, LD- long duration

The mean GY of the MRQ74 PLs with a single and with different combinations of *qDTYs* (QTL class – A, B, C, D, E, F, and G) together with the recipient parent (no QTL class – X) are presented in Table 2; Fig. 5. In general, the mean GY of the MRQ74 PLs with either a single or different combinations of *qDTYs* in all RS and NS trials was higher than that of MRQ74. Under RS, the mean GY for the MRQ74 PLs was significantly higher than that of MRQ74 for both long and medium duration lines.

**Table 2:** QTL class mean comparisons for grain yield in kg ha^−1^ under drought stress (stress) and irrigated control (non-stress) in MRQ74 as the recipient parent in trials conducted during the 2013 and 2014 dry seasons

QTL class label	QTLs combinations	2013 - medium duration		2013 - long duration		2014 - long duration	
		*NS*	*RS*	*NS*	*RS*	*NS*	*RS*
A	*qDTY*_*2.2*_ *+ qDTY*_*3.1*_ *+ qDTY*_*12.1*_	3272.27 a	951.84 d	4544.57 ac	817.15 cb	5244.69 a	191.04 a
B	*qDTY*_*12.1*_ *+ qDTY*_*3.1*_	3986.11 bc	984.3 d	4825.65 cb	1210.89 d	5790.29 b	369.56 ab
C	*qDTY*_*12.1*_ *+ qDTY*_*2.2*_	4388.46 c	559.49 b	4799.92 cb	1129.34 d	7622.61 c	264.89 ab
D	*qDTY*_*2.2*_ *+ qDTY*_*3.1*_	3926.70 bc	639.04 b	4090.89 a	652.74 b	5550.93 ab	563.68 b
E	*qDTY*_*12.1*_	3980.007 bc	591.76 b	5159.42 c	875.56 c	5809.43 b	493.41 cb
F	*qDTY*_*3.1*_	3690.20 b	829.87 c	4176.96 a	660.57 b	5409.14 a	340.68 ca
G	*qDTY*_*2.2*_	3713.37 ba	718.59 bc	4348.73 ab	1163.43 d	5388.17 ab	960.36 d
MRQ74	NO QTL	4332.34 bca	56.73 a	4034.43 ac	90.41 a	5105.24 ab	115.07 ab
F- value		3.20	10.01	4.82	10.87	17.09	4.42
*p*-value		0.0002	<0.0001	0.0001	<0.0001	<0.0001	0.0003

Means followed by the same letter are not significantly different

Among the MRQ74 PLs with single *qDTY*, PLs with *qDTY*_*12.1*_ (class E) and *qDTY*_*2.2*_ (class G) performed considerably well across NS and RS trials as compared to PLs with *qDTY*_*3.1*_ (class F). Between the PLs of the two classes- E and G, PLs with *qDTY*_*2.2*_ (class G) yielded higher than PLs with *qDTY*_*12.1*_ (class E) under RS and PLs with *qDTY*_*12.1*_ (class E) yielded higher than PLs with *qDTY*_*2.2*_ (class G) under NS across all seasons. PLs with *qDTY*_*3.1*_ (class F) was a moderate yielder under both stress and non-stress.

Among the MRQ74 PLs with two *qDTYs, qDTY*_*12.1*_ *+ qDTY*_*2.2*_ (class C) and qDTY_12*.1*_ *+ qDTY*_*3.*1_ (class B) were good yielder under NS as well as RS. However, PLs with qDTY_12*.1*_ *+ qDTY*_*3.*1_ (class B) yielded higher than PLs with *qDTY*_*12.1*_ *+ qDTY*_*2.2*_ (class C) in RS with yield difference being statistically significant only in 2013 medium duration RS trial. PLs with *qDTY*_*2.2*_ *+ qDTY*_*3.1*_ (D) were moderate yielders under NS and RS.

In general, the PLs with three QTL combinations (*qDTY*_*2.2*_ *+ qDTY*_*3.1*_ *+ qDTY*_*12.1*_) did not show significant yield advantage over the PLs with two QTL combinations under both NS and RS except for higher yield of PLs with *qDTY*_*2.2*_ *+ qDTY*_*3.1*_ *+ qDTY*_*12.1*_ (class A) over PLs with *qDTY*_*2.2*_ + *qDTY*_*3.1*_ (class D) in the 2013 RS trials. Under NS, most of the QTL combination PLs yielded close to or better than that of MRQ74. Under RS, the mean yields of the PLs were higher in both years and significantly different from that of MRQ74 in 2013.

### Performance of promising drought-tolerant pyramided lines

The performance of 11 promising drought-tolerant BC_1_F_5_ MRQ74 PLs is presented in Table 3. The recipient parent MRQ74 produced 95 kg ha^−1^ GY in RS trials. DTF of selected MRQ74 PLs varied greatly in both RS (84 to 102 days) and NS trials (81 to 97 days). However, the DTF of the selected lines were in general lower than that of MRQ74, indicating that the MRQ74 PLs flowered earlier than the recipient parent. Plant height of MRQ74 PLs was not much different from MRQ74. The GY of selected MRQ74 PLs ranged from 5158 to 7452 kg ha^−1^ in the NS trials and from 1195 to 3642 kg ha^−1^ in the RS trials during the 2014 DS. Under NS trials in 2013 and 2014, most of the QTLs combinations PLs yielded similar or higher than the yield of MRQ74 except for the yield of PLs IR 98010-202-274-1-1, IR 98010-202-68-B-2, IR 98010-410-62-1-2 in 2013 NS.

**Table 3:** QTL content, days to 50 % flowering (DTF), plant height (PHT), and grain yield (GY) of the most stable lines across two seasons of lowland drought stress and non-stress conditions

PLs	*qDTY*_*12.1*_	*qDTY*_*3.1*_	*qDTY*_*2.2*_	DTF				PHT				GY (kg ha^−1^)			
				2013		2014		2013		2014		2013		2014	
				*NS*	*RS*	*NS*	*RS*	*NS*	*RS*	*NS*	*RS*	*NS*	*RS*	*NS*	*RS*
IR 98008-103-445-1-1	√	-	√	76	81	85	87	92	74	95	65	5340	2930	6517	1249
IR 98010-126-376-1-1	-	√	-	94	98	94	102	82	64	76	49	4307	1038	7284	1203
IR 98010-126-708-1-3	√	-	-	99	94	81	82	67	73	82	68	4337	3328	5158	2554
IR 98010-126-708-1-4	√	-	-	99	94	82	81	67	73	86	69	4337	3328	6865	3642
IR 98010-126-708-1-5	√	-	-	99	94	84	88	67	73	92	66	4337	3328	5408	2063
IR 98010-126-846-1-2	√	-	-	90	95	91	88	89	68	90	67	5788	1512	6936	1831
IR 98010-202-274-1-1	-	-	√	76	78	81	84	77	63	82	61	3800	1557	5630	1573
IR 98010-202-68-B-2	√	√	-	76	77	82	86	78	60	79	58	3504	2419	5238	1195
IR 98010-410-62-1-2	√	-	-	79	84	97	98	70	50	71	56	3175	795	7452	1394
IR 99616-44-94-1-1	√	√	-	100	89	82	84	81	66	88	67	5206	907	5586	2165
IR 99616-44-94-1-2	√	√	-	100	89	82	85	81	66	90	69	5206	907	5732	2051
MRQ74	-	-	-	91	95	96	105	83	60	78	55	4044	95	5104	169

*NS* non-stress, *RS* reproductive stage drought stress

## Discussion

### Selection of parents

The selection of the recipient and donor parents is the most important step in developing superior rice varieties. In this study, the high quality Malaysian rice cultivar MRQ74 was used as recipient parent as it carries desired traits such as long grain, good aroma, short plant type, and moderate to high tolerance to multiple pests and diseases. MRQ 74, the recipient parent in the study is resistant to blast, tungro and moderately resistant to bacterial blight, brown plant hopper and sheath blight in Malaysia. It possessed filled grain of 84.63 %, milled rice recovery of 68 %, milled grain length of 6.62 mm, milled grain width of 1.88 mm, length/width ratio of 3.56, thousand grain weight of 24.46 gm. It has amylase content of 27, gel consistency of 70 and was rated as highly scented rice with scent score of 2 with soft texture of cooked rice (Asfaliza et al. 2008; Asfaliza et al. 2012; Rafii et al. 2014).

The selection of major-effect QTLs to be used in breeding programs is also critical in achieving the needed yield advantage under drought. The QTL × genetic background and QTL × environment interactions both induce inconsistency in estimated QTL effects (Bernardo 2008). To tackle these interaction effects, pre-breeding lines with major and consistence effects of *qDTYs* combining high yield potential and good yield under drought were used as *qDTY* donor parents. All three *qDTYs* used in this study were identified to produce good yield in severe RS conditions individually in different mapping populations or genetic backgrounds (Bernier et al. 2007; Venuprasad et al. 2009). The use of pre-breeding lines or lines with improved plant type possessing a major-effect QTL is the key to the successful implementation of the QTL introgression program (Ye and Smith 2010; Swamy and Kumar 2013).

### Development of drought tolerant MRQ74 PLs using stepwise MAB technique

An economic yield advantage of at least 1000 kg ha^−1^ of yield under drought is desired to provide an economic yield benefit to farmers (Kumar et al. 2014). MRQ74 PLs provided a yield advantage of 1000 kg ha^−1^ or more which proves that marker assisted QTL pyramiding of major-effect *qDTYs* through backcross breeding is an effective strategy to increase rice yield under drought.

Marker assisted pyramiding enables breeders to introgress two or more QTLs that control the same or different traits associated with biotic and abiotic stresses in plants. The stepwise genotyping technique followed in this study led to a dramatic reduction in the number of plants to be genotyped which in turn led to a significant reduction in the cost of genotyping. MAB with a stepwise screening technique has been previously applied to select genotypes with desirable genes/QTLs by reducing the number of selected individuals in each step (Servin 2004; Sreewongchai et al. 2010). Together with the stepwise screening technique, this study also used three types of markers: (i) peak markers which are tightly-linked to the QTL region; (ii) flanking markers to eliminate the linkage drag; and (iii) background markers for fast recovery of the recipient parent’s genetic background (Neeraja et al. (2007).

The selection process started with foreground genotyping where the *qDTY* loci were monitored by three peak markers, with one peak marker for each *qDTY* locus. In the foreground selection, the peak markers reported in an earlier study, namely RM520 (Venuprasad et al. 2009) and RM511 (Bernier et al. 2007), were used to confirm the presence of *qDTY*_*3.1*_ and *qDTY*_*12.1*_, respectively. However, the reported peak marker RM236 for *qDTY*_*2.2*_ (Swamy et al. 2013) was replaced with RM12460 (the closest polymorphic marker in the *qDTY*_*2.2*_ region) as RM236 was monomorphic among MRQ74 and the *qDTY*_*2.2*_ donor. In order to increase selection efficiency and to reduce linkage drag, three to six flanking markers for each *qDTY* were used in the recombinant selection (Hospital and Charcosset 1997; Neeraja et al. 2007). The use of flanking markers for recombinant selection may help recover all the important traits of the recipient parent. In the case of traits like drought tolerance, where gene-linked markers are not available, the identification of peak markers and flanking markers in the recipient background within the QTL region is necessary at the initiation of a MAB program.

Donor fragments of approximately 7.5 Mbp for three drought yield QTLs, *qDTY*_*2.2*_ (2.3 Mbp)*, qDTY*_*3.1*_ (1.7 Mbp), and *qDTY*_*12.1*_ (3.5 Mbp), that were introgressed into MRQ74 crosses represent about 1.7 % of the genome. However, with this size of introgressed fragments, linkage drag might still occur and affect the phenotype of the plants with *qDTYs* although all *qDTYs* were successfully introgressed. Dixit et al. (2012) reported the fine-mapping of *qDTY*_*2.2*_ and *qDTY*_*12.1*_ regions and found that introgression of the fine-mapped region could minimize the undesirable linkage of the *qDTY* in future MAB programs.

MAB has been an effective and efficient strategy in crop improvement as it speeds up and simplifies the selection process especially for complex traits (Sirithunya et al. 2004; Collard et al. 2005; Dayteg et al. 2007). Several researchers successfully pyramided QTLs/genes for multiple disease resistance to provide a broader spectrum of resistance than those conferred by a single QTL/gene (Castro et al. 2003; Zhang et al. 2006; Pinta et al. 2013; Sreewongchai et al. 2010).

The total numbers of equally spaced SSR markers across the genome used for background selection in this study was 48 and 61–72 in the F_1(3)_ and BC_1_F_1_ generations, respectively. The selected BC_1_F_1_ MRQ74 PLs represent 82 to 90 % of the recipient genome. According to Septiningsih et al. (2008), one marker every 5 Mb or 20 cM or a total of four to nine well-distributed markers per chromosome depending on the length of the chromosome is adequate to monitor the donor introgressions. Similarly, Servin and Hospital (2002) reported that two to four markers on a chromosome of 100 cM with the same distance between two markers could provide adequate coverage of the genome in a backcross program through MAB simulation study.

### Severity of reproductive stage drought stress trials

In the 2013 DS, the MRQ74 PLs were grouped into medium and long duration PLs and RS treatment was imposed at different dates for each group to minimize the variation in flowering time among segregating populations. A mean yield reduction of more than 70 % indicated that in both seasons the level of drought stress achieved was severe. Lafitte (2003) reported that a 50 % reduction in GY is compulsory in identifying true drought-tolerant lines. However, Kumar (2008) did not detect any response of selection in RS trials that produced a yield reduction of 56% and recommended a screening protocol that could reduce the mean yield by at least 65 % in RS trials compared to irrigated control trials.

### Agronomic performance of MRQ74 PLs under RS and NS conditions

A significant variation on DTF among MRQ74 and their PLs across two DS was observed more in the RS trials compared to the NS trials. The mean DTF under RS was higher than in NS, indicating that drought stress delayed flowering time. Similar results were observed by several researchers (Atlin et al. 2006; Jongdee et al. 2006; Vikram et al. 2011; Ghimire et al. 2012; Dixit et al. 2014). In addition, the high *H* for DTF observed in this study was also found by several earlier studies (Sedeek et al. 2009; Sohrabi et al. 2012). A moderate *H* for plant height under RS as against a high *H* under NS has resulted in variation in plant height under RS caused by a differential reduction in the height of drought tolerant and susceptible genotypes. The medium to high *H* for GY under both RS and NS trials recorded in this study has also been reported earlier (Swamy and Kumar 2013).

MRQ74 is shown to be extremely sensitive to severe RS condition as its relative yield reduction ranged from 86 to 99 %. Its PLs, however, were less affected by RS. Most of the MRQ74 PLs flowered earlier than the recipient parent under both RS and NS, which may have been caused by the linkages of *qDTYs* with early flowering (Kumar et al. 2014). Most of the drought-tolerant donors flower early and alleles for DTF and GY under drought have been reported to be linked in some studies (Kumar et al. 2014).

### Performance of different combinations of qDTY

MRQ74 PLs with either single or different combinations of *qDTYs* produced higher yields compared to the recipient parent under RS, indicating the positive effect of the introgressed *qDTYs* under RS condition. Single QTL PLs with *qDTY*_*12.1*_*, qDTY*_*2.2*_*,* and *qDTY*_*3.1*_ showed increased yield under drought in the background of MRQ74 as predicted in the mapping populations against the Vandana, IR64, and Swarna genetic backgrounds. This validates the effectiveness of the *qDTY*_*12.1*_*, qDTY*_*2.2*_*,* and *qDTY*_*3.1*_ QTLs in increasing yield under drought against different genetic backgrounds. The PLs with *qDTY*_*2.2*_ showed a stable and high effect across all the seasons. Earlier, Swamy et al. (2013) had shown the high yield advantage imparted by *qDTY*_*2.2*_ in the IR64 background. In the IR64 background, PLs with two QTLs namely *qDTY*_*2.2*_ *+ qDTY*_*4.1*_ have been released as varieties in India, Nepal, and Myanmar. This is the first study that showed the effectiveness of *qDTY*_*2.2*_ in genetic backgrounds other than IR64. The study also confirmed the effect of *qDTY*_*12.1*_ in lowland drought in diverse genetic backgrounds. Bernier et al. (2007) and Dixit et al. (2012) have reported the effect of *qDTY*_*12.1*_ in the Vandana genetic background in upland while Mishra et al. (2013) identified its effect in lowland in the Sabitri background. This study validated the effect of *qDTY*_*12.1*_ in lowland in the MRQ74 background.

Under the NS condition across the two DS, the MRQ74 PLs with two *qDTYs* (*qDTY*_*2.2*_ + *qDTY*_*12.1*_) gave the highest mean GY compared to MRQ74 PLs with other *qDTY* combinations. However, *qDTY*_*3.1*_ + *qDTY*_*12.1*_ was not significantly different from *qDTY*_*2.2*_ + *qDTY*_*12.1*_ in two of the three cases under NS and did well under RS. This indicates that *qDTY*_*12.1*_ *+ qDTY*_*3.1*_ is appropriate in the MRQ74 background to achieve yield increases under RS as well as to maintain high yields under NS. However, PLs with only *qDTY*_*3.1*_ and PLs with *qDTY*_*2.2*_ *+ qDTY*_*3****.****1*_ both produced moderate yields under both conditions across the two years.

These results showed that *qDTY*_*12.1*_ and *qDTY*_*2.2*_ either as single or as a combination with other *qDTYs*, contributed to major effect when introgressed into MRQ74. The results also clearly imply the necessity to identify *qDTY* combinations with positive interaction against different genetic backgrounds for appropriate yield increase under drought. MRQ74 PLs with two *qDTY* combinations stood as top performers for grain yield in severe RS trials as well as in NS trials. This supports the findings of Swamy et al. (2013) that a combination of at least two *qDTYs* is necessary to achieve at least 1000 kg ha^−1^ yield advantage under RS. PLs with three *qDTY* combinations were expected to perform better than PLs with a single and with two *qDTY* combinations but the results are contrary in this study. Similarly, Swamy et al. (2013) reported that lines with two and three QTL combinations performed better than lines with four QTL combinations. This result indicates (i) a non-linear interaction between multiple *qDTYs* and (ii) the presence of differential synergistic relationship between *qDTY* combinations (Dixit et al. 2012; Swamy et al. 2013).

### Performance of identified improved drought tolerance PLs

Eleven BC_1_F_5_ MRQ74 PLs with yield advantages of 1009 to 3473 kg ha^−1^ under RS and with yield higher than MRQ74 under NS trials were classified as promising drought tolerance PLs. Five MRQ74 PLs produced more than 2000 kg ha^−1^ of yield under severe RS while another set of five MRQ74 PLs also gave more than 1000 kg ha^−1^ of yield advantage under NS condition. Among the selected lines, PLs with the single *qDTY*_*12.1*_ were most often the best performers compared to other *qDTY* combinations followed by PLs with two *qDTYs* (*qDTY*_*3.1*_ + *qDTY*_*12.1*_). Comparison among all selected MRQ74 PLs indicated that 82 % carried *qDTY*_*12.1*_, 36 % carried *qDTY*_*3.1*_, and only 18 % carried *qDTY*_*2.2*_ either as a single *qDTY* or a combination with other *qDTYs.* This suggests that *qDTY*_*12.1*_ works well in the background of MRQ74.

Furthermore, MRQ74 PLs with *qDTY*_*12.1*_ either as a single or a combination with other two lowland *qDTYs* showed a greater effect on GY under NS, which indicates that *qDTY*_*12.1*_ is superior to other *qDTYs* and is consistent across the planting season. The higher yield advantage of selected PLs over the yield advantage observed in class analysis of PLs with different QTL combinations/variable yield advantage of lines selected from the one observed in class analysis may have resulted from the capture of positive interactions between QTLs and the genetic background during drought phenotyping in the process of selection. For any MAB/MAS program for complex traits like drought, where less is known about the QTL x QTL, QTL x environment and QTL x genetic background interactions, positive interactions can be captured through a combination of markers with selection for yield under standardized managed RS screens (Kumar et al. 2014).

## Conclusions

The study has successfully developed BC_1_F_5_ MRQ74 PLs that provided a yield advantage of 1009 to 3473 kg ha^−1^ over their recipient parent under RS and with an acceptable yield potential under NS, indicating that *qDTY* pyramiding through stepwise MAS strategy is an efficient and effective approach to improve drought tolerance in rice. Appropriate QTL combinations providing high yield advantage under RS in the MRQ74 background were identified. The study proposes the combination of MAB with appropriate phenotyping as a suitable approach for complex traits like drought to select plants with higher yield advantage under RS.

## Methods

### Plant materials

Three drought-tolerant lines, IR77298-14-1-2-10 possessing *qDTY*_*2.2*_, IR81896-B-B-195 with *qDTY*_*3.1*_, and IR84984-83-15-18-B with *qDTY*_*12.1*_, were used as donor parents to transfer three *qDTYs* in the recipient parent MRQ74 using QTL pyramiding technique (Servin 2004; Sreewongchai et al. 2010) followed by one time backcrossing and selfing thereafter.

### Marker assisted breeding and genotyping

DNA marker work was conducted at the Molecular Marker Application Laboratory of the Plant Breeding, Genetics, and Biotechnology Division of IRRI. Fresh leaves from all lines were collected and freeze-dried. DNA extraction of leaf samples was carried out using modified CTAB protocol (Murray and Thompson 1980). A total of 125 SSR markers linked to three *qDTY* regions (foreground selection) and an additional 711 SSR markers distributed in the whole rice genome which are unlinked to *qDTY* regions (background selection) were tested in a polymorphism survey. However, only three peak markers and an additional 13 flanking markers were found polymorphic in the three *qDTY* regions and were thus used in foreground selection in every generation. The peak markers linked to the three *qDTY* regions on chromosome 2, 3, and 12 were RM12460, RM520, and RM511, respectively (Table 4). All SSR markers were assayed on the rice population as described by Panaud et al. (1996). The polymerase chain reaction products were separated in 6 % or 8 % non-denaturing polyacrylamide gel electrophoresis. The DNA fragments were then stained with SYBR Safe and visualized with UV trans-illuminator. The DNA profiles from such markers were scored in comparison with their parents. Plant selection in each generation was dependent on a number of plants that carried the target regions. Stepwise marker assisted selection and phenotyping technique was applied to select and advance the appropriate plants to decrease the number of samples in every generation.

**Table 4:** Details on drought yield QTLs (*qDTYs*) used in the marker assisted breeding

Recipient	Donor	NIL	Ecosystem	*qDTY* name	Chromosome	Interval	Peak marker	a	R^2^
IR64	Adaysel	IR77298-14-1-2-10	Lowland	*qDTY*_*2.2*_	2	RM154-RM279	RM12460	14	6
Swarna	Apo	IR81896-B-B-195	Lowland	*qDTY*_*3.1*_	3	RM520-RM16030	RM520	30	27
Vandana	Way Rarem	IR84984-83-15-18-B	Upland	*qDTY*_*12.1*_	12	RM28048-CG29430	RM511	47	33

*NIL* near isogenic line, additive effect compared to trial mean (a, in percentage), phenotypic variance (R^2^, in percentage)

Source: Swamy and Kumar (2013)

### Evaluation of pyramided lines

After genotyping, 206 BC_1_F_3_ (separated between medium and long duration PLs) and 230 BC_1_F_5_ MRQ74 PLs with different *qDTYs* and their combinations were selected based on similarity of morphological characteristics with MRQ74. These 206 BC_1_F_3_ and 230 BC_1_F_5_ MRQ74 PLs were evaluated together with their recipient and donor parents in the field under lowland RS and NS conditions during the 2013 and 2014 DS. Field-based phenotyping experiments were conducted at the IRRI in lowland transplanted conditions (IRRI, Los Banos, Philippines, 14^0^ N 121^0^E, 21 m above sea level). Lowland refers to field experiments conducted under flooded, puddled, and transplanted conditions. The term RS is used in experiments where drought stress was imposed during the reproductive stage of the crop while NS is used in experiments where drought stress was not imposed (control condition). In total, six lowland experiments (three under RS and three under NS) were conducted using this population. MRQ74 PLs and MRQ74 were evaluated in an alpha lattice design with two replications (Patterson & Williams 1976) in plot sizes of two rows of 5 m length at 25 cm × 25 cm spacing. Missing hills were replanted by stock seedlings within 10 days of transplanting. A total of 50 plants were maintained in each plot. Inorganic fertilizers (N:P:K) were applied at the rate of 90:30:30 kg ha^−1^. The post emergence herbicide Sofit (pretilachlor 0.3 kg a.i. ha^−1^) was applied 4 days after transplanting; hand weeding was also done for weed control. For the control of stem borers and other insects, Furadan (carbofuran 1 kg a.i. ha^−1^) at 5 days after transplanting and Cymbush (cypermethrin 1 kg a.i. ha^−1^) at 16 days after transplanting were applied. To control snails, the molluscicide Bayluscide (niclosamide 0.25 kg a.i. ha^−1^) was also applied.

For the RS experiments, the fields were irrigated to maintain soil moisture at field capacity or above for four weeks after transplanting. RS was imposed four weeks after transplanting by draining water from the field. Perforated PVC pipes were placed in 100 cm soil depth at four different points of the field. Daily water table depth after stress initiation in the RS experiments was also recorded. Data on daily rainfall, daily maximum and minimum temperature, and relative humidity for the trial period were recorded. The fields were allowed to dry until the soil cracked and the surface was completely dry. The fields were re-irrigated when the check varieties as well as 70 % of the entries showed severe leaf rolling. When the water table reached below 100 cm and remained such for about three weeks, irrigation was given by flash flooding and the fields were drained again after 24 h (Venuprasad et al. 2007) to impose a second cycle of the stress. Parching water table was measured from all the pipes every day after draining the field until crop reached 50 % maturity. For the NS experiments, 5 cm water level was maintained in the fields throughout the crop season until drain before harvesting. The NS experiments were conducted to obtain the data on the performance of PLs under the control condition so as to select lines combining high yield under NS and good yield under RS conditions.

### Data collection

Data on days to 50 % flowering, plant height, and grain yield were recorded from all experiments. DTF were recorded as the number of days from sowing till the day on which 50 % of the plants had flowering tillers. Plant height (in cm) of three plants from each plot was measured at maturity from ground level to the tip of the tallest tiller and was averaged for analysis. Lines with DTF between 75–90 days were grouped as medium duration and those with more than 90 days DTF as long duration lines. GY from each plot was harvested at physiological maturity, dried to a moisture content of 14 %, and weighed. The plot data was then converted to kg ha^−1^ and used for analysis.

### Statistical analysis

Data from each trial were analyzed using PB Tools v1.1.0. The genotype means were estimated using a linear mixed model that considered replications and blocks within replications as random effects while the genotypes were considered as fixed effect. Broad-sense heritability (*H*) of the traits measured in the RS and NS conditions was calculated as

$$ H = \frac{s_g^2}{s_g^2+{s}_e^2/r} $$

where ${s}_g^2$ is the genotypic variance, ${s}_e^2$ is the error variance, and *r* is the number of replications.

### Selection of drought-tolerant pyramided lines

BC_1_F_5_ MRQ74 PLs with different *qDTY* combinations that produced more than 1000 kg ha^−1^ under RS and an acceptable GY under NS were identified during the 2014 DS. These identified MRQ74 PLs were classified as drought-tolerant MRQ74 PLs. A total of 17 BC_1_F_5_ MRQ74 PLs were classified as the most promising as they produced more stable and consistence GYs across two DS (2013 DS and 2014 DS).

### Class analysis

#### QTLs combinations class analysis

The performance *y*_*ijkl*_ of the *j*^*th*^ genotype nested within the *i*^*th*^ QTL class in the *l*^*th*^ block within the *k*^*th*^ replicate is modelled as follows:

$$ {y}_{ijkl}=\mu +{r}_k+b{(r)}_{kl}+{q}_i+g{(q)}_{ij}+{e}_{ijkl} $$

where *μ* is the population mean, *r*_*k*_ is the effect of the *k*^*th*^ replicate, *b*(*r*)_*kl*_ + *q*_*i*_ is the effect of the *l*^*th*^ block within the *k*^*th*^ replicate, *q*_*i*_ is the effect of the *i*^*th*^ QTL, *g*(*q*)_*ij*_ is the effect of the *j*^*th*^ genotype nested within the *i*^*th*^ QTL, and *e*_*ijkl*_ is the error (Knapp, 2001). The effects of the QTL and genotypes within the QTL are considered fixed while the replicate and blocks within replicate effects are considered random.

## Abbreviations

*qDTYs*: drought yield QTLs
DS: dry season
*H*: heritability
IRRI: International Rice Research Institute
KDML 105: Khao Dawk Mali 105
REML: linear mixed model analysis
MAB: marker assisted breeding
MMAL: molecular marker application
NS: non-stress
PBGB: plant breeding, genetics, and biotechnology
PLs: pyramided lines
QTLs: quantitative trait loci
RS: reproductive stage drought stress
SSR: simple sequence repeat
